# Cross-Modal Perception of Noise-in-Music: Audiences Generate Spiky Shapes in Response to Auditory Roughness in a Novel Electroacoustic Concert Setting

**DOI:** 10.3389/fpsyg.2018.00178

**Published:** 2018-02-20

**Authors:** Kongmeng Liew, PerMagnus Lindborg, Ruth Rodrigues, Suzy J. Styles

**Affiliations:** ^1^School of Art, Design and Media, Nanyang Technological University, Singapore, Singapore; ^2^Graduate School of Human and Environmental Studies, Kyoto University, Kyoto, Japan; ^3^Soundislands, Singapore, Singapore; ^4^Raffles Arts Institute, Raffles Institution, Singapore, Singapore; ^5^Division of Psychology, School of Social Sciences, Nanyang Technological University, Singapore, Singapore

**Keywords:** noise, human–computer interaction, cross-modal perception, auditory roughness, computer music, multimedia

## Abstract

Noise has become integral to electroacoustic music aesthetics. In this paper, we define noise as sound that is high in auditory roughness, and examine its effect on cross-modal mapping between sound and visual shape in participants. In order to preserve the ecological validity of contemporary music aesthetics, we developed *Rama*, a novel interface, for presenting experimentally controlled blocks of electronically generated sounds that varied systematically in roughness, and actively collected data from audience interaction. These sounds were then embedded as musical drones within the overall sound design of a multimedia performance with live musicians, Audience members listened to these sounds, and collectively voted to create the shape of a visual graphic, presented as part of the audio–visual performance. The results of the concert setting were replicated in a controlled laboratory environment to corroborate the findings. Results show a consistent effect of auditory roughness on shape design, with rougher sounds corresponding to spikier shapes. We discuss the implications, as well as evaluate the audience interface.

## Introduction

At the turn of the 20th Century, the Italian futurist composer Luigi Russolo proposed that with the advent of noisy machinery, audiences in contemporary European societies had begun to lose their sensitivity to music. Composers had to expand their musical boundaries, by breaking free of centuries of tradition in music; of the excessive attention to Pythagorean ratios in musical harmony. In 1913, his manifesto the Art of Noise declared that:

“*Musical art aims at the shrillest, strangest and most dissonant amalgams of sound… we are approaching noise-sound*” (Russolo, 1913/1967).

A century later, noise has entrenched its place in the world of contemporary art and music, working its way into a wide range of music from experimental composers (e.g., Karlheinz Stockhausen and Iannis Xenakis), to pop musicians (e.g., The Beatles) and audio–visual artists (e.g., Ryoji Ikeda). Musicologists and philosophers have long debated the distinction between music and noise [notably, Theodore Adorno (1970/1984) stated that music may share the same origins as noise but is differentiated by its rationality and organization]. However, some fields of electroacoustic music actively manipulate sounds generally thought of as ‘noisy’ as structural elements of their audio design. Such uses of art-noise make it difficult to support such binary distinctions between noise and music. Rather, for the purposes of this paper, we treat noise as sound that is high in auditory roughness. This paper focuses on the perceptual effects of noise as an element of sound design in the context of a live electroacoustic concert, using dynamic, audience-based responses to cross-modal stimuli as a probe.

### Auditory Roughness as Noise

Noise has been described as sound that is “lacking agreeable musical quality,” “noticeably unpleasant” (Merriam-Webster, 2017) or “unwanted” (Truax, 1999), and these definitions bear resemblance to the effects of auditory roughness in its association with annoyance (McDermott, 2012), and consistently high ratings of unpleasantness (Plomp and Levelt, 1965; Fritz et al., 2009; Bonin et al., 2016; Lahdelma and Eerola, 2016). Furthermore, this approach follows established practices in sound-quality engineering research, where auditory roughness is often used as a measure of environmental noise (e.g., Gonzalez et al., 2003; Wang et al., 2013), and has been shown to correlate directly with ratings of noisiness (Sato et al., 2007). As such, in this paper, we assume equivalence between noise and roughness: the rougher a sound, the noisier it is.

Auditory roughness was first coined by Hermann von Helmholtz in 1885, in reference to the raspy, harsh, and buzzing sound caused by tones in narrow intervals (Vassilakis, 2005), that cannot be physiologically differentiated in a harmonic relationship. This is due to the inability of the human auditory system (basilar membrane) to differentiate simultaneous close frequencies in a range known as the critical band (Campbell and Greated, 1987; Vassilakis, 2005). Auditory roughness can also be understood through its acoustic mechanisms: constant, rapid amplitude fluctuations in the sound spectrum in the range of 20–200 Hz (Parncutt, 1989), resembling an auditory sensation of buzzing. This can be a result of direct amplitude modulation (AM) of carrier frequencies, which is a form of external interference resulting in rapidly changing loudness levels (Fastl and Zwicker, 2007), found in, for example, a combustion engine of an old car. Auditory roughness can also be created by phase interference from narrow harmonic intervals, which naturally occurs in any sound with complex spectral properties (e.g., speech and musical instruments). In pure sinusoidal wave dyads, slight mistuning in the second frequency causes the relationship of the two waves to be slightly out of phase. As a result, the sound waves interfere with each other producing the characteristic amplitude fluctuations associated with roughness. Auditory roughness can thus be modeled by calculating the difference in amplitudes of these two frequencies against the critical band (Zwicker, 1961), and in complex sounds, the roughness from prominent pairs of formants in the sound spectrum are summed to give an overall score of auditory roughness (MacCallum and Einbond, 2008; Bernardes et al., 2014).

### Cross-Modal Perception of Sound, Noise, and Shapes

Lab-based studies investigating cross-modal perception have shown pervasive links between the auditory and the visual domain. For example, participants prefer to match high pitched sounds to small, spiky shapes, bright colors, and stimuli with a high position in visual space (for a review, see Spence, 2011), effects that are even seen in young children (Mondloch and Maurer, 2004; Walker et al., 2010). Few studies have investigated cross-modal perception for noisy stimuli, or the acoustic property of roughness. One important study showed that participants listening to sounds of the same pitch, but with different waveforms, also show a cross-modal matching preference, with participants preferring to match tones produced on a square wave to a more-angular shape than tones carried on a smooth sinusoid (Parise and Spence, 2012). This example is illustrative, as one acoustic property of the square wave is a higher level of acoustic roughness. In our own laboratory investigations, we have seen a correlation between the roughness of auditory stimuli and the preference for verbal descriptions of spikiness and roughness, and for particular visual objects differing in the spikiness of their edges [for preliminary report, see (Liew et al., 2017)]. Furthermore, Hung et al. (2017) found that similar kinds of sound-shape mappings for linguistic stimuli “bubu” and “kiki” occurred pre-consciously. One possible driver of such linguistic effects could be the formant structure of the vowels /i/ and /u/ (Morton, 1977; Lockwood and Dingemanse, 2015; Styles and Gawne, 2017). As auditory roughness can be measured from the acoustic properties of vowels with different formants, it is possible that roughness contributes to the effect. In the context of music, the link between auditory roughness (or noise) and the visual domain has not been systematically employed, although it has been implied in the immersive audio–visual installations of noise-heavy audio–visual sound-artists such as Ryoji Ikeda.

Given that cross-modal processing is often implicit, or even unconscious (Ramachandran and Hubbard, 2001; Evans and Treisman, 2010; Parise and Spence, 2012), a more systematic understanding of the effects of multimedia stimuli will bring novel perspectives to composers, artists, and audio–visual designers. In our attempt to understand specific effects of noise in music, we apply the findings of our earlier laboratory study, in an actual concert setting. In this paper, we describe *Rama*, an interactive musical interface for testing audience responses to noise in a musical context, in multimedia performance environments, via collaborative audience shape-design. We report here the use of *Rama* in a live contemporary music performance, the collective results of the concertgoers, as well as a laboratory-based replication.

In the present paper, we investigate the impact of noise with controlled auditory roughness levels on visual object preference, in two different artistic scenarios – a live concert setting, and a controlled lab replication. In the concert setting, a live, improvised musical performance was accompanied by musical drones, which comprised of synthesized digital sounds with predetermined levels of noise (auditory roughness). Audience members then collectively controlled the appearance of an interactive visual projection using their smartphones in response to the overall sound. In the lab setting, individuals controlled the appearance of the visual object while listening to a recording of the live concert. Our unconventional method of involving audience interaction in a performance environment presents us with an opportunity to investigate sound-shape mappings in a novel context. In comparison to past literature on cross-modal mapping, our experimental method allows for testing in situations of high ecological validity in the contemporary arts space, and gives the participant (audience) agency in deciding a precise extent of sound-shape mapping. Thus, our outcome variable serves two functions with a similar outcome: First, in replicating previous laboratory results, auditory roughness should robustly predict cross-modal sound-shape mappings even when disguised as noise in a live interactive contemporary arts context. Second, this demonstration also allows us to test whether the multimedia performance environment is suitable for quantifying audiences’ cross-modal experience of auditory stimuli, with the use of an interactive, visual object, generated by live digital interaction between audience members.

## Designing a Multimedia Performance Environment in Max

**FIGURE 1:**
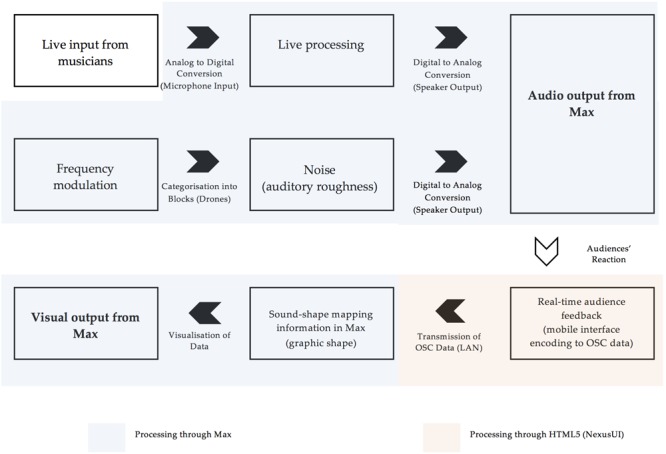
A conceptual flow of the elements of *Rama*.

With the intent of collecting real-time data from audience members, we designed a multimedia performance environment in [Max/MSP (6. 1. 6), 2016], named *Rama*. To this end, the different experimental conditions were embedded within the time-based structure of the performance, to which audience members could provide real-time feedback through a LAN-based interface on their smartphones. This mobile interface enabled access to data collection, which consequently affected the generated visuals of the performance in real time. **Figure 1** shows an illustration of the flow of elements in *Rama*.

In order to control visual and sonic information simultaneously, Max was used as the main programming language, and NexusUI (Taylor et al., 2014), a JavaScript toolkit running on HTML5, was used to program a LAN-based mobile interface for data collection. Auditory roughness was controlled through embedding a frequency modulation (FM) synthesis patch within the main *Rama* framework. These allowed us to control roughness through FM synthesis (see section 3.2 Stimuli) and formed the experimental levels which differed on roughness (Modulation Index) and pitch (Carrier Frequency). MacCallum and Einbond’s (2006) Real-Time Analysis of Sensory Dissonance patch, which estimated auditory roughness according to Parncutt’s (1989) models, was also incorporated to provide quick visual confirmation of generated roughness levels during playback. Detailed screen captures of the Performance interface can be found in the Open Science Framework repository for this article: https://osf.io/axz4s.

*Rama* was designed for an interactive audio–visual performance, with predetermined experimental elements subsumed within the structure of the work. This resulted in three key design features: Firstly, since the audience experience was intended as an aesthetic experience (and not as a laboratory experiment), auditory roughness levels were smoothly integrated into the sound design of the composition, not as overwhelmingly distinct elements of the piece. Secondly, blocks of noise were incorporated into the performance at predefined points in the composition, and the roughness levels of these noise blocks were strictly controlled. Lastly, live data collection allowed the audience to collectively control the shape of a projected object as part of the interactive experience, and this data stream was available for offline analysis of the sound/shape mapping preferences of the audience.

Since the aesthetic experience was essential to the ecological validity of the experiment, the roughness conditions were embedded in the sound design of the performance, behind live musicians and other aesthetic/musical devices, in the form of electronic (musical) drones. A four-channel input with live processing (multichannel delay, sample and hold) was used to enable live input from performing musicians on any unspecified instrument into the electronic soundscape of the piece. Overlaid on this performance, at pre-determined points in the composition, the Max *urn* object allowed the experimentally controlled roughness drones to be presented as sequential blocks of noise following a random order. A buffer was also created for three channels to allow spontaneous recordings and time-stretched or inverted playback to create several drone-like sounds. These added a compositional coherence to the aesthetic structure of the performance, by creating similarity between the experimental blocks of noise (also presented as drones), and the general sonic structure of the composition. The buffer also allowed for control of the frequency range throughout the composition. This meant that all levels of pitches in the experimental noise blocks could be partially masked, regardless of instrument selection for any given performance. For example, if the selection of instruments for a given performance included only high-pitched instruments like violins and flutes, the controlled playback would allow low-pitch sounds to be electronically generated from these instruments in the performance, thereby integrating seamlessly with lower-pitched roughness blocks, and preventing these blocks from standing out too much.

**FIGURE 2:**
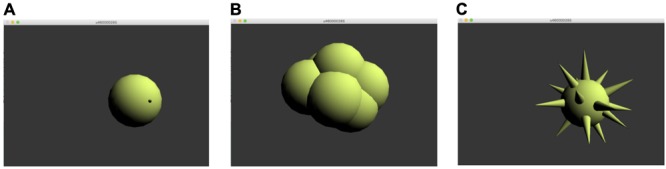
**(A)** default object, **(B)** object at maximum curviness, and **(C)** object at maximum spikiness.

In order to examine audiences’ visual preferences in response to the different blocks of noise, a 3D rendered circular object was generated in Max through the *jit.gl.gridshape* object, as illustrated in **Figure 2**. The object’s shape could change on a continuum between spiky and curvy, depending on real-time audience feedback through a LAN-based smartphone interface. Audience members saw a controller with buttons for “more curvy” and “more spiky.” At any point in the performance, audience members could push one of these buttons to change the shape of the projected image accompanying the performance.

The animation of the graphic was made by creating several overlapping conical and spherical structures hidden inside the base circular object. Audience ratings were converted to a single metric of average shape (from curvy to spiky) in real time, and determined how much of each shape projected above the surface of the base sphere. The resultant shape of the object was therefore time-locked to specific points in time in the performance, allowing for a detailed quantification of an averaged shape according to audience responses to the controlled noise-blocks within the performance.

### Graphical Interface for Audience Feedback

To incorporate the feedback from audience participants into the shape of the onscreen graphic, a simple interface was needed to link audience involvement with the larger *Rama* Max patch. Ultimately, an interactive web interface accessible by smartphones was created on HTML5 with elements from NexusUI to encode audience responses as OSC information, which could then be imported in real time from the *udpreceive* object in Max. Participants all joined a single wireless network as the host device (concurrently running the main *Rama* patch), in order for each participant’s feedback to be downloaded automatically.

A QR code with the link embedded enabled quick access to the site. At this stage, audience members were asked to consent to participating in the study before being redirected to the interface. The interface included ‘buttons’ for two primary responses: “More Curvy” or “More Spiky.” Each click on the “More Curvy”/“More Spiky” button would increase/decrease the size of the desired attribute (spikes or spheres) in the 3D object by 2% of the maximum spiky/curvy size (1 point out of a bidirectional 100 point scale). If audience members agree on the ‘best’ shape to accompany a particular sound, the choices should result in consensus on the object’s shape. Following our previous lab-based investigations, we predicted that noisier sounds (sounds in the higher roughness conditions), would have a higher agreement in spikiness, resulting in a spikier shape, and vice versa.

Through the support of a group of musicians at the South-Eastern Ensemble for Today’s and Tomorrow’s Sounds (SETTS), *Rama* was performed at a concert at the Visual Arts Centre, Singapore in March 2017. Rama was scheduled to be both the opening and the closing item. For this performance, the ensemble comprised of four musicians: pianist, bassoonist, flutist, and violist.

## Hypothesis

In order to investigate the effects of noise (high auditory roughness) on cross-modal sound-shape mapping with the *Rama* interface, we conducted two experiments. The first was an examination of sound-shape mapping in the context of an electroacoustic music concert, prioritizing ecological validity over laboratory control. The second was a laboratory replication, where participants were played an audio recording of the live concert. With the *Rama* interface, we present a novel response method for cross-modal sound-shape mapping by having participants control the exact extent of the spikiness/curviness for each sound, in real time.

Our hypothesis is that noise, defined by auditory roughness, drives audio–visual mappings to visual spikiness, and that this psychoacoustic property functions independently from other known correlates of spikiness, such as pitch height. We therefore predicted that in both experiments, varying degrees of noise (auditory roughness) should predict participants’ design of object, regardless of the pitch of the drones, with noisier sounds from the high roughness blocks corresponding to spikier object designs, and less noisy sounds from the low roughness blocks corresponding to curvier object designs.

## Experiment 1

### Methods

#### Participants

Audience members at a live electroacoustic music event were invited to participate. The concert was held at the Visual Arts Centre in Singapore in March 2017. The number of active participants was estimated to be 16 (the LAN-based system pooled data from all participants, meaning that individuation of results was not possible). To maintain the simplicity of audience participation in an arts context, no demographic information was collected. This experiment received approval from the Institutional Review Board of Nanyang Technological University. All participants gave informed consent before participating in the study.

#### Stimuli

The interface, *Rama*, was designed in the Max programming environment, with additional interfaces designed in HTML 5 (see section “Designing a Multimedia Performance Environment in Max”). Musicians were requested to create an improvised piece constrained by the following loose instructions: play notes a major third apart from each other at any point in time, and to avoid harmonic clashes. Combined with the electronics, we were able to build a spectrally full sound through live signal processing and delay algorithms. This sonic structure effectively masked the experimental nature of a series of 16 sequentially presented 20-s blocks of noise, presented in a random order. These blocks were generated through FM synthesis, in a manner similar to Liew et al. (2017). Carrier frequencies (base frequencies) were set at four predetermined pitches: A0 (55Hz), A1 (110 Hz), A2 (220Hz), and A3 (440Hz). At each specified pitch, four levels of roughness were set according to MacCallum and Einbond’s (2006) roughness estimations: high roughness (1.0), mid roughness (0.5), low roughness (0.01) and no roughness (0). The consistent variation in roughness levels was achieved through the adjustment of the modulation index for each level, which varied the intensity of harmonic bands across the frequency spectrum (Hass, 2001). The harmonicity of the FM synthesis was kept consistent at 6.7, which meant that the harmonic series of the synthesized sound did not follow typical frequency ratios. Amplitude was kept constant for all synthesized sounds. The *Rama* patches are available in the Open Science Framework repository for this project: https://osf.io/axz4s.

The music itself was structured into 16 different segments, each 20 s long. The segments differed in the background electronic blocks of noise that were sustained in a drone-like manner throughout the length of the segment. The order of the presentation of blocks was determined by a random process. Due to a technical error in real-time audio rendering, the first six stimuli resulted in silent presentation, but the remaining ten stimuli were presented at the correct intensity. Although this resulted in a smaller number of experimental blocks, the random order of presentation, and the systematic variation in roughness provided sufficient data for analysis of the real-time results in a concert setting. The recording of the live concert performance including *Rama* synthesis is available in the Open Science Framework repository for this project: https://osf.io/axz4s.

#### Procedure

At the start of the concert, audience members were guided through the LAN login procedure, and were instructed to push the buttons “more spiky” and “more curvy” to control the shape of the visual object during the performance. Following a short demonstration, four musicians (piano, flute, viola, and bassoon) from the SETTS Ensemble, a Singaporean professional new music ensemble, performed an improvised piece, accompanied by *Rama’s* synthesized elements, designed by the first author. All electronic sounds were projected by a stereo speaker setup with Fostex PM0.4n speakers and a Fostex PM Sub8 subwoofer. Audience members were invited to collectively reshape the computer graphic in a manner fitting to the music. All graphics were presented on a 27-inch HD monitor, positioned between the performers and the audience at the audiences’ eye-level. Based on live feedback from each participant, via a LAN webpage connected to the main *Rama* interface through Open Sound Control (OSC) data, the visual object with varying shape (curvy to spiky) (see section “Designing a Multimedia Performance Environment in Max”, **Figure 2**) was displayed on a monitor. The displayed shape was determined by the collected response from the audience, from a minimum score of -50 (curvy) to a maximum of 50 (spiky).

#### Data Handling and Analysis

Participant response data were transformed to a continuous scale from 0 to 1 for the analysis. Time and final shape information (outcome variable) were captured and stored every second, allowing for convenient mapping to auditory roughness condition in encoding at a rate of 20 data points for 20 s of audio for each auditory roughness condition. The first five data points were discarded for each condition, allowing time for participants to adjust to the onset of each sequence. The resultant shape information was then recorded as an average of participant feedback on a continuous scale of 0 to 100, with 100 being extremely spiky, and 0 being extremely curvy. The object returned to a neutral (50) state at the onset of every block.

For data analysis, the 10 stimuli were divided into two groups, according to ‘lower’ and ‘higher’ roughness, along an arbitrary representation of noise. The cut off was set at roughness = 0.5 according to MacCallum and Einbond’s (2006) model: Hence, ‘Lower Roughness’ consisted of no roughness and low roughness stimuli (0.01), while the ‘Higher Roughness’ consisted of the noisier sounds: the mid roughness (0.5) and high roughness (1.0) stimuli. This two-level analysis was used to compensate for the unequal number of stimuli at the four initial roughness levels, after dropout. As the analysis was planned for stimulus-level analysis, the sample size (*N* = 10) was too small for the application of parametric analyses. As such, a non-parametric Mann–Whitney *U* test was conducted in SPSS.

### Results and Discussion

**FIGURE 3:**
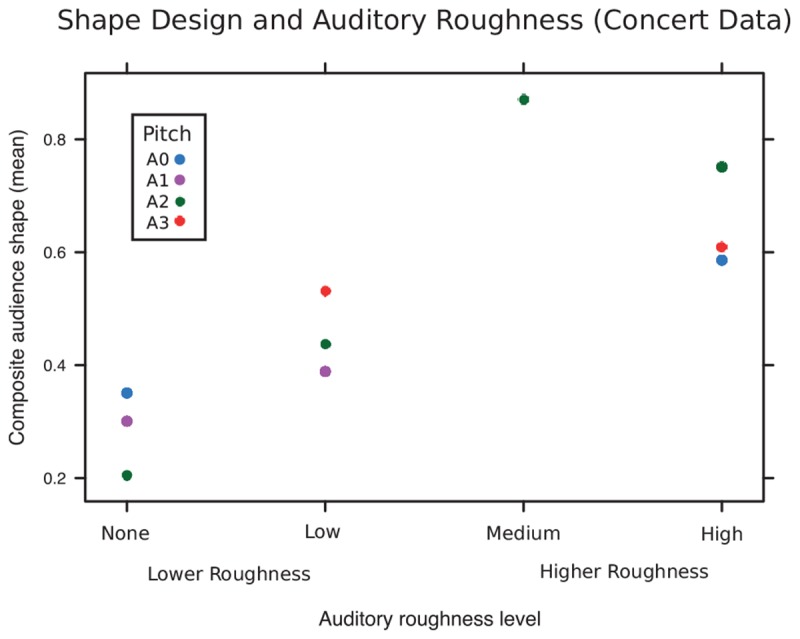
Collaborative audience shape-design in Experiment 1 (Live Concert setting), for each block of noise, showing different levels of roughness, and pitch.

The Mann–Whitney *U* test revealed that the medians for the Lower Roughness group (*N* = 4) and Higher Roughness group (*N* = 6) were different (*U* = 0.67, *p* = 0.048). Participants created a significantly spikier object for musical segments with auditory stimuli from the Higher Roughness group (mid and high auditory roughness levels), and a significantly curvier objects for segments with low/no Roughness. Composite shape design is shown in the four roughness categories in **Figure 3**.

Despite its limitations, the data collected during a live concert revealed an effect of auditory roughness (noise) on shape design, corroborating our earlier findings that sound-shape mapping is influenced by auditory roughness (Liew et al., 2017). As such, the *Rama* interface was successful in collecting real-time audience data. However, the study had several limitations. To have ecological validity, aesthetic experience was prioritized over laboratory control, and this carried several inevitable implications. Firstly, to ensure that the experimental elements did not ‘pop out’ from the general performance context, the combinations of noise and improvisation were not as discretely controlled as would be possible in a non-performance context – indeed, as the musicians were improvising, the nature of their performance may have differed in the context of different kinds of noise, as the performers incorporated the noise into their improvised performance. These interactions between experimentally controlled ‘noise’ and live improvised musical performance may have created self-reinforcing feedback between the instrumental and synthesized elements of the composition: That is to say, segments with experimentally controlled high-roughness may have become even rougher, and vice versa. Secondly, due to the uncontrolled nature of the live concert scenario, not all audience members decided to take part in the interactive element of the performance. This may have been due to the size of the display (a large digital monitor), which did not deliver a fully immersive visual experience, or to individual differences in audience interactivity preferences (only a small proportion of audience members chose to participate). Thirdly, due to large number of simultaneous processes, *Rama* had a slight system lag, which resulted in delayed timestamps (a 1-s time interval in *Rama* corresponded to an actual interval of approximately 1.075 s). Finally, *Rama* was performed in a somewhat niche electro-acoustic concert, meaning the audience was somewhat unusual in their level of musical knowledge and engagement. This ‘expert’ audience may show a different pattern of audio–visual mapping preferences when compared to a less-musically experienced audience. To further examine whether the effect of auditory roughness on shape design extends beyond the live audience participation context, we conducted a replication of this study in a laboratory setting.

## Experiment 2

### Methods

#### Participants

Participants (*N* = 27, males = 11, 1 unidentified) were undergraduates aged 18–24 (*M* = 22.5, *SD* = 1.7) recruited from Nanyang Technological University for course credit. This experiment received approval from the Institutional Review Board of Nanyang Technological University. All participants gave informed prior consent before participating in the study.

#### Stimuli

Participants listened to a recording of the performance from the live concert (recorded on a Zoom H6n device in stereo. WAV format, sample rate: 48 kbps, bit depth: 24). As such, stimuli remained the same as Experiment 1 (note that the laboratory replication included only those blocks of noise that were rendered correctly in the live performance, hence, the same small number of roughness conditions were missing). The recording of the live concert performance including *Rama* synthesis is available in the Open Science Framework repository for this project: https://osf.io/axz4s.

#### Procedure

Unlike Experiment 1, the *Rama* interface was modified to only facilitate audio playback instead of real-time rendering of audio and stimuli. Participants listened to the audio playback in the psychology laboratories with a computer and Sennheiser HD280 headphones. Instead of the two-button LAN-based smartphone interface used in experiment one, participants dragged a bidirectional slider on the modified *Rama* Max interface, allowing individual participants to shape the object (on a continuum from spiky to curvy), as they desired. Data was collected based on the position of the slider, on a continuous scale from 0 to 1. Note that in Experiment 1, the resultant shape was based on a collection of audience responses, but in Experiment 2, each participant designed the shapes independently. One additional difference was implemented for this solo playback condition – the shape did not ‘reset’ at the beginning of each noise block. We believed this would allow solo participants to focus on the micro-structure of the composition, without being primed to pay attention to the blocked design.

#### Data Handling and Analysis

Following Experiment 1, participant response data were transformed to a continuous scale from 0 to 1 for analysis. Time and final shape information (outcome variable) were captured and stored every second, allowing for convenient mapping to auditory roughness condition in encoding at a rate of 20 data points for 20 s of audio for each auditory roughness condition. The first five data points were discarded for each condition, allowing time for participants to adjust to the onset of each sequence. The resultant shape information was then recorded as an average of participant feedback on a continuous scale of 0–1, with 1 being extremely spiky, and 0 being extremely curvy. Unlike Experiment 1, the object did not return to a neutral state at the onset of every block.

Data analysis was conducted using linear mixed-effect modeling with the lme4 package (Bates et al., 2015) in R (R Core Team, 2017). *p*-values were subsequently calculated with Satterthwaite approximation to degrees of freedom with the lmerTest package (Kuznetsova et al., 2016). Random slope models were used, with the random effects of participant and stimulus, and fixed effects of auditory roughness (high, medium, low, none) and pitch (A0, A1, A2, A3). Observed power was simulated through the simr package (Green and MacLeod, 2016). Pirateplots were conducted in R, following Phillips (2016).

### Results and Discussion

**FIGURE 4:**
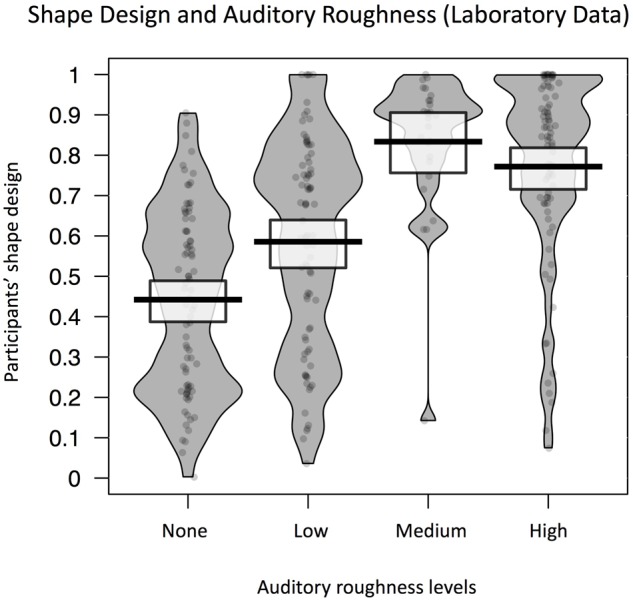
Pirateplot showing overall pattern of individual shape design in Experiment 2 (solo lab setting), shown separately for different roughness levels. Shape design is significantly predicted by auditory roughness. The grey shaded area represents the distribution of participants’ shape design for each level of roughness, and the average lines and white boxes represent means and 95% highest density intervals (HDIs).

**Figure 4** shows the average shape design for blocks of noise at each level of auditory roughness. A likelihood comparison of models revealed the best fitting model to be with the fixed effect of auditory roughness only. Auditory roughness significantly predicted participants’ shape design [*b* = 0.1, *SE* = 0.03, *t*(7.8) = 3.4, *p* = 0.01]. **Figure 4** is a Pirateplot of shape design in the different roughness conditions. *Post hoc* power simulations revealed observed power = 0.65 (effect size, *f*^2^ = 0.11). This represents a small effect size, in a low-powered sample (probably due to a small sample size). However, these data represent proof-of-principle for the influence of auditory roughness on shape characteristics in audio/visual design.

Experiment 2 therefore replicates the direction of results found in Experiment 1: whether in a lab, or in a live concert setting, auditory roughness predicts the shape designed by individuals and groups of participants. Rougher, noisier sounds correspond to spikier graphics. Additionally, when examining the Pirateplot of results for Experiment 2 (**Figure 4**), the bimodal distribution of shape for the low and no-roughness conditions suggests that when roughness is low, cross-modal sound-shape mapping is variable, and may depend on a variety of other auditory features. Conversely, when auditory roughness crosses the threshold of 0.5 into the medium and high ranges, it strongly informs participants’ design of a spiky shape. It is likely that in such acoustically complex situations, participants’ map visual shape via a complex integration of various auditory cues. It is only when auditory roughness is high that perhaps attention is focused on the rough stimulus, in turn mapping on to spiky visual shapes. Such an interpretation would be consistent with Bonin and Smilek (2016) findings on the automatic cognitive interference effect of auditory roughness (from inharmonic chords) in attentional and memory tasks. Interestingly, even though numerous previous studies have shown that higher pitches are typically paired with sharper shapes (e.g., Boyle and Tarte, 1980; Marks, 1987; Walker et al., 2010; Shang and Styles, 2017), in the current study, the roughness characteristics of noise predicted of shape design more strongly than the acoustic feature of pitch. This suggests a role for the relatively unexplored dimension of auditory roughness in future investigations into sound-shape matching, and cross-modal correspondence in general.

**FIGURE 5:**
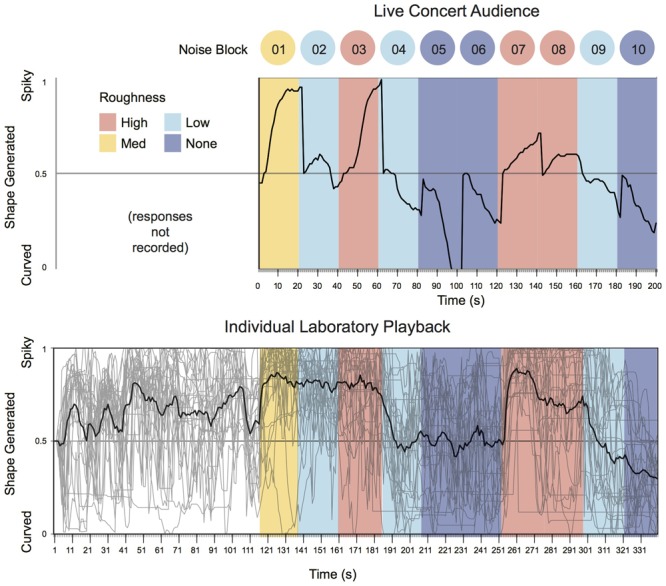
A time-series visualisation of aggregated participant (audience) responses between Experiments 1 (Live Concert Audience) and 2 (Individual Laboratory Playback). Thick line indicates group mean. Thin lines indicate individual responses (Experiment 2 only). Note that the coding of time stamps in Experiment 1 suffered from a slight lag meaning that each ‘second’ was actually slightly larger than 1 s. Scales have been equated for visual comparison.

**FIGURE 6:**
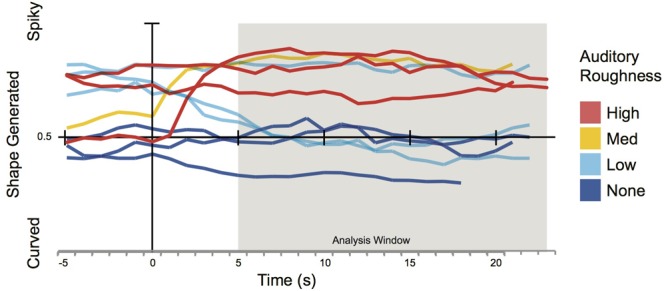
Time-aligned average responses to each block of noise in Experiment 2 with Roughness type shown. 0 s indicates onset of noise block. Analysis window was from 5 s to end of noise block.

In an exploratory examination of the time-series data (**Figures 5, 6**), it is notable that the collaborative shape-generation in the concert setting of Experiment 1, and the mean of the solo shape generation in Experiment 2 have substantially similar profiles. This coherence is in line with the theoretical predictions of the current project, but all-the-more surprising given the differences in audience composition, and other aspects of the implementation (e.g., the shape defaulting to neutral at the beginning of each noise block in the live concert setting, but not in the solo lab-setting). Some differences are also evident. For example, we find that the audience response system in Experiment 1 has a more dynamic profile, reaching the maximum extent of each design at the very end of the noise blocks – perhaps indicating that audience members with a preference for more extreme shapes persist in voting often to help the ‘group’ achieve their preferred extent – resulting in a wider dynamic range. By contrast, the average of individual participants’ responses (Experiment 2) plateaus earlier within each block of noise – suggesting more systematic response patterns overall. However, the individual traces in Experiment 2 show that individuals’ response patterns are highly dynamic and in-the-moment. This implies that participants are highly attentive to the microstructure of the performance, with unique patterns of responding; the effects of the noise are only evident in the aggregate.

This exploratory analysis allows additional novel insights into individual variance in response to complex combinations of noise in music. Firstly, when a group ‘collaborates’ to create a shape, the individual variance is effectively ‘smoothed’ to show the group’s combined efforts, however, this shape likely represents the preferences of more persistent audience members, rather than a stable amalgam of all audience members’ preferences. Conversely, solo-viewing paradigms allow more detailed investigations of multisensory responses to the microstructure of a musical composition. Future investigations of this kind can bear these parameters in mind when designing interactive response interfaces for multisensory investigations.

## General Discussion

### Cross-Modal Sound-Shape Mapping

Despite the main focus of the live performance being the musical improvisation by live quartet, audience members were able to translate the systematically controlled roughness stimuli in the different blocks of noise that formed part of the composition into shape-matching decisions in the visual domain. This demonstrates that even overlaid across an improvised musical composition (in both live, and controlled playback of the concert recording), noise, in the form of auditory roughness, can influence multimodal decisions about matching in the visual domain. This finding adds to the robustness of sound-shape mapping between auditory roughness and spikiness in the visual domain: In our previous laboratory-based studies investigating the acoustic properties of Western and Chinese traditional musical instruments, and FM synthesis, we have seen that both the rated dissonance and the measured auditory roughness of different instrument notes predict the edge characteristics of shape choices between pairs of complex 3D objects (Liew et al., 2017). Here, we not only see that auditory roughness predicts spikiness in a shape generation paradigm, but also that the roughness incorporated into the sound design as noise elements within an improvised musical piece, is sufficiently salient to trigger similar audio–visual matches.

In contrast to our previous results, here we found that participants systematically created spiky shapes for the noisier blocks of mid- and high-roughness auditory stimuli, but a more mixed pattern of responses for no- and low-roughness stimuli. This may be due to the greater degree of control over the precise extent of sound-shape mapping allowed for with the *Rama* interface, as compared to the limited measurement used in our previous study: a two-alternative forced choice between a spiky shape and a rounded shape. This explanation would imply that the current findings are more reflective of the effects of auditory roughness in the visual domain. Alternatively, it may be due to the properties of the improvised musical content during the performance, which may have been more ‘available’ for participants’ attention during the low- and no- roughness stimuli.

Despite these differences between our current and previous findings, these experiments nevertheless demonstrate that cross-modal effects can be observed in the context of contemporary musical performance using novel audience participation techniques, especially when high levels of noise are present in the composition. As a sizeable portion of electronic music today revolves around the aesthetic of noise, we demonstrate that there are subtle effects of noise that go beyond an auditory listening experience to influence other sensory modalities. The fact that noise in music is able to influence the conceptualisation of shape, opens up possibilities for future experimentation in other types of influence that noise may convey. At this stage, however, it is unclear whether the cross-modal perception of noise through roughness is driven by affective properties of spectrally complex sounds, or whether the roughness itself modulates low level cross-modal processing, as both spiky, angular shapes and high roughness sounds are similarly associated with negative valence (Palumbo et al., 2015; Lahdelma and Eerola, 2016). However, since this is the first study of its kind into the relationship between noise and shape, we are hesitant to attribute the effect to a single affective/hedonic cause. In particular, we are cautious that there may be a low level perceptual analog between visual stimuli with high fluctuations in their edge characteristics (spiky/rough) and acoustic stimuli that exhibit high fluctuations in their spectral characteristics (‘noisy’ sound). It is possible that the neural coding of both types of noise may be sufficiently similar that the modalities share a sensory structure (i.e., they ‘match’), and the affective outcome of each type of noise is a consequence of their shared structural properties. Alternatively, it is possible that auditory roughness is affectively unpleasant, and visual roughness is independently unpleasant, and the ‘match’ is due to their shared affective status, not to any shared sensory substrates. At the present time, there is insufficient evidence to determine whether affect causes the match between modalities, or is a consequence of the match between modalities that share sensory processes. More detailed investigations into neural coding and conscious percept of different kinds of noise would be needed to tease apart these possibilities.

However, given the current findings, multisensory research will benefit from more nuanced investigations of into low-level acoustic properties of complex sounds along these lines, and by including auditory roughness as a dimension of future interest. In the context of computer music, such findings can help composers and artists deliver a more immersive artistic message through pieces that are grounded in a unified perceptual experience.

### An Evaluation of Rama as an Interface for Quantifying Audience Interaction

*Rama* was introduced as an interface for a multimedia performance environment that allowed collection of audience response data. One of the objectives of this paper was to evaluate its performance in relation to the results of the experiments. The direction of the effects observed in the concert were in agreement with the findings of our earlier laboratory study, demonstrating that the property of auditory roughness is replicable in an electroacoustic concert setting with higher ecological validity than the previous laboratory-based design. *Rama* therefore successfully met the following design criteria: (a) live performance of auditory ‘drones’ with experimentally controlled roughness levels, and pre-defined pitch attributes, performed as a ‘fifth instrument’ in a concert setting; (b) online collation of audience responses in real time; and (c) data collection allowing the quantification of participants’ responses. One limitation of the current *Rama* system is the way that individual devices were not trackable, hence audience responses were consolidated into a single data stream, meaning that individual responses could not be compared. Furthermore, the participation pathway was somewhat cumbersome, which may have led to low motivation to participate in the concert setting.

Nevertheless, *Rama* provides proof of concept for integrated arts-science performances where interactive aesthetic experiences are paired with experimentally controlled data collection. Audience response interfaces like *Rama* have the potential to bring a new dimension into perception and cognition research. With the ability to consolidate participant responses outside of the laboratory and into an environment like a concert hall or an art gallery, researchers can develop testing methods suited to the sampling of situations with high ecological validity (e.g., Kawase and Obata, 2016). However, more care should be given to integrate audience participation with the research design. Given that a platform like Max allows for easy integration of various plug-ins, future studies could explore other effects of noise and roughness with measures, such as motion tracking, that are less intrusive to the aesthetic experience of audience members.

## Conclusion

Although music theorists are divided about how best to characterize noise in music, contemporary music aesthetics have established perceptually ‘noisy’ elements as integral to the sound design of many genres of music. When noise is characterized by the acoustic property of roughness, our experiments show that noise has impacts on cross-modal aesthetic experience, and biases peoples’ preference for particular visual shapes. In particular, people tended to generate shapes with longer spikes for movements of the improvised composition that were accompanied by drones with high roughness. As our experiments sampled audience/listener responses while listening to an electroacoustic music piece that featured controlled levels of background noise in real time, we demonstrate an ecological validity to this phenomenon beyond the laboratory.

## Materials and Data Availability

The audio recording of the concert, precise task instructions from the lab-based task, and the raw data are available in an Open Science Framework repository, along with the Max patches used to create Rama: https://osf.io/axz4s/.

## Ethics Statement

This study was carried out in accordance with the recommendations of IRB of the authors’ University with online informed consent from all subjects. All subjects gave online informed consent in accordance with the Declaration of Helsinki. The protocol was approved by the IRB of the authors’ University.

## Author Contributions

KL, PL, and SJS conceived and designed the experiments, analyzed the data and wrote the paper. RR and KL performed the experiments. KL designed the sounds and programmed *Rama*. KL and SJS designed the visuals for *Rama* and prepared the figures. RR contributed materials.

## Conflict of Interest Statement

The authors declare that the research was conducted in the absence of any commercial or financial relationships that could be construed as a potential conflict of interest.
